# Performance and Prognostic Relevance of Lymph Node Assessment by One-Step Nucleic Acid Amplification Assay in Rectal Cancer: A Multicenter Study

**DOI:** 10.3390/cancers17132141

**Published:** 2025-06-25

**Authors:** Qing Liu, Sandra Lopez-Prades, Karmele Saez de Gordoa, Maite Rodrigo-Calvo, Mireia Garcia, Juan Ruiz Martin, Angel Romo, Ignacio Pinilla, Jordi Tarragona, Begoña Otero Alen, Jordi Camps, Ivan Archilla, Miriam Cuatrecasas

**Affiliations:** 1 August Pi i Sunyer Biomedical Research Institute (IDIBAPS), 08036 Barcelona, Spain; liuq_0607@126.com (Q.L.); slopezp@recerca.clinic.cat (S.L.-P.); saezdegord@clinic.cat (K.S.d.G.); mtrodrigo@clinic.cat (M.R.-C.); jordi.camps@uab.cat (J.C.); 2 Faculty of Medicine and Health Sciences, University of Barcelona, 08036 Barcelona, Spain; 3 Pathology Department, Hospital Clinic Barcelona, 08036 Barcelona, Spain; garcia01@clinic.cat; 4 Pathology Department, Hospital Universitario de Toledo, 45004 Toledo, Spain; jmartin@sescam.jccm.es (J.R.M.); aromo@sescam.jccm.es (A.R.); 5 Pathology Department, Hospital Universitario Severo Ochoa, 28911 Madrid, Spain; inapinilla@live.com; 6 Pathology Department, Hospital Universitari Arnau de Vilanova, 25198 Lleida, Spain; jtarragona@gss.cat; 7 Pathology Department, University Hospital Complex A Coruña (CHUAC), 15006 A Coruña, Spain; begona.otero.alen@sergas.es; 8 Centro de Investigación Biomédica en Red de Enfermedades Hepáticas y Digestivas (CIBERehd), 28029 Madrid, Spain; 9 Cell Biology and Medical Genetics Unit, Department of Cell Biology, Physiology and Immunology, Faculty of Medicine, Autonomous University of Barcelona (UAB), 08193 Bellaterra, Spain

**Keywords:** rectal carcinoma, lymph node metastases, staging, diagnosis, prognosis, OSNA

## Abstract

Background/Objectives: Lymph node metastases (LNM) undetected by standard hematoxylin and eosin (H&E) have been associated with unfavorable prognosis in colorectal cancer. The One-Step Nucleic Acid Amplification (OSNA) assay has demonstrated superior sensitivity in detecting LNM compared to H&E. We aimed to assess the performance of OSNA in detecting LNM, as well as its prognostic value in rectal cancer (RC) patients. Methods: Lymph nodes (LNs) of patients from 15 centers were analyzed by both H&E and OSNA. The total tumor load (TTL) was defined as the sum of cytokeratin 19 mRNA copies/µL in all LNs from a surgical specimen, using a threshold of 250 copies/μL for OSNA positivity. Cox proportional hazard regression was used to assess the effect of TTL ≥ 250 or 6000 copies/μL on cancer-specific survival (CSS) and recurrence-free survival (RFS), with Firth’s method applied to account for low event rate. Results: A total of 97 RC patients were included. Of these, 84 patients were eligible for survival analysis. The sensitivity and specificity of OSNA, compared to H&E, were 91.7% and 84.7%, respectively. TTL ≥ 6000 versus <6000 copies/μL was related to worse CSS and RFS. When dividing TTL into three groups: ≤250, 250–6000, and >6000 copies/μL, only TTL ≥ 6000 copies/μL was significantly associated with worse CSS and RFS. Conclusions: The OSNA assay is highly sensitive for detecting LNM in RC patients. A TTL of ≥6000 copies/μL could identify a subset of RC patients with worse CSS and RFS who might benefit from adjuvant treatment or intensive surveillance.

## Simple Summary

The presence of micrometastases in lymph nodes (LNs) has been associated with unfavorable prognosis in patients with colorectal cancer. A fraction of patients with stage III disease may be under-staged due to the limited sensitivity of conventional hematoxylin and eosin (H&E) analysis in detecting lymph node metastases (LNM). The One-Step Nucleic Acid Amplification (OSNA) assay has demonstrated superior performance in detecting LNM and holds prognostic significance. We aimed to assess the performance of the OSNA assay in detecting LNM and its prognostic value in rectal cancer (RC) patients. LNs were analyzed by both standard H&E and the OSNA assay. We concluded that the OSNA assay is highly sensitive for detecting LNM in RC and allows identification of a subset of RC patients with worse cancer-specific survival and recurrence-free survival who might benefit from adjuvant treatment or intensive surveillance.

## 1. Introduction

In 2022, GLOBOCAN estimated colorectal cancer (CRC) as the third most prevalent malignancy with 1,926,425 new cases globally, and the 2nd cause of death by carcinoma [1]. CRC etiology is multifactorial, including genetic, environmental, and lifestyle factors. Whereas most CRC occur in people over 50 years old, early-onset CRC among adults younger than 50 years has nearly doubled since 1990, which may be mainly related to lifestyle factors, environmental exposures and diet [2]. The 5-year relative survival rate for stage I CRC in the US is about 92%, and for stage IIA and IIB is 87% and 65%, respectively. Surprisingly, the 5-year survival rate for stage IIIA and stage IIIB are slightly higher than Stage II, being 90% and 72%, respectively, while for stage IIIC it is 53%, and 12% for stage IV [3]. Most CRC slowly progresses from precursor lesions such as adenomatous polyps or sessile serrated lesions, which enable a time frame for screening and detecting early-stage carcinomas. CRC screening programs have significantly altered the landscape of CRC by increasing its diagnosis at an early-stage (stages I-II; pT1-4 N0), which exceeds 70% among all screened CRC patients. In addition, CRC screening programs have achieved a reduction in the mortality from this disease and are cost-effective [4,5,6]. This new scenario has also expedited surgical treatment, being associated with less invasive surgeries and reduced rates of emergent surgeries and complications. All these have ultimately contributed to an improvement in both overall survival (OS) and disease-free survival (DFS) [4,7].

The presence of lymph node metastases (LNM) represents one of the most important prognostic factors in CRC, as it is closely related to disease recurrence and survival, and it is pivotal in determining the use of adjuvant chemotherapy (aCT) following surgery [8]. As defined by the Union for International Cancer Control (UICC), occult tumor cells in LNs comprise micrometastases (MMs) and isolated tumor cells (ITCs). These small-sized LNM could pose a potential prognostic hazard in early-stage CRC patients. Previous meta-analyses revealed that MMs, rather than ITCs, in histologically negative LNs (pN0), were associated with poor prognosis in CRC patients [9,10]. Another meta-analysis demonstrated that molecularly detected occult metastases were associated with worse OS, DFS, and cancer-specific survival (CSS) in node-negative CRC patients [11]. Moreover, in a prior study, the results indicated that nodal micro- and macro-metastases had comparable median OS (82 vs. 69 months), with a non-significant shorter trend than node-negative patients (118 months) [12].

Conventional LN staging with hematoxylin and eosin (H&E) only analyses a 2–5 μm thick section of each LN, which accounts for approximately 0.06% of the LN tissue [13]. Thus, a small fraction of the LN is assumed to be representative of the entire LN [13]. In fact, postoperative aCT is routinely recommended for stage III CRC patients and some high-risk stage II patients. A subset of stage III CRC patients might be under-staged due to the limited sensitivity of standard H&E in detecting LNM, thereby being undertreated and compromising their long-term survival [14,15]. This is evidenced by the fact that approximately 20–25% of stage I/II CRC patients may develop disease recurrence following curative-intended surgical resection [16,17]. Thus, there is a survival paradox for stage IIB/C patients, showing higher recurrence rates and worse prognosis compared to stage IIIA patients [18,19]. The performance of additional H&E sections increases the detection rate of occult metastases from 3.8% to 6.3 and 11.8% with one, two, and five sections analyzed by H&E, respectively [20]. However, it is not feasible to incorporate LN multi-sectioning into routine practice. Therefore, in early-stage CRC, there is an unmet clinical need to develop more sensitive and reliable techniques for a more accurate LN analysis and staging.

Various molecular techniques have been proposed to improve the detection of small LNM, such as the use of immunohistochemistry (IHC) or reverse transcription polymerase chain reaction (RT-PCR) [21]. Most IHC-based studies used antibodies against different cytokeratins to detect the presence of carcinoma cells in the LNs, since the latter are naturally devoid of epithelial cells. Those studies using RT-PCR to detect tumor cells in LNs amplified fragments for different RNA markers, i.e., KRT19 (CK19), CEACAM5 (CEA), forkhead box A1 (FOXA1), SAM-pointed domain containing ETS transcription factor (SPDEF), tumor-associated calcium signal transducer 2 (TACSTD-2), mucin 1 (MUC1), and MGB1 [22]. Nevertheless, these techniques are laborious, costly, and time-consuming. Hence, a rapid, highly sensitive, and cost-effective diagnostic method is needed. The one-step nucleic acid amplification (OSNA) assay is a quantitative, fast, and automated PCR-based technique for detecting cytokeratin 19 (CK19) mRNA using the reverse-transcription loop-mediated isothermal amplification (RT-LAMP) method. Yamamoto and colleagues demonstrated that OSNA was superior to H&E in detecting LNM, equivalent to a histopathological multilevel sectioning at 2-millimeter intervals [23]. In a prior study, the OSNA method up-staged pN0 CRC patients diagnosed with H&E, being 2.0%, 17.7%, 12.5%, and 25% for stages I, IIA, IIB and IIC, respectively [24]. The OSNA assay has been shown to be a cost-effective procedure for the management of stage II CRC patients [25]. Furthermore, some studies have demonstrated the prognostic value of the OSNA results in CRC, which are related to worse DFS and OS [26,27]. Nevertheless, its role in rectal cancer (RC) patients remains to be explored.

Colon cancer (CC) and RC have been synonymously called CRC in all fields of research and clinical practice. However, some studies have demonstrated different origins based on their anatomy, biology, metabolism, genetic characteristics, treatment, and prognosis [28,29,30,31,32]. Historically, RC demonstrated a poorer prognosis than CC, irrespective of the use of multimodal therapy [33,34]. Recently, this trend is shifting, since the long-term survival of RC patients appears to be even better than that of patients with CC due to a series of improvements in treatment strategies, mainly including the introduction of total mesorectal excision (TME) and neoadjuvant chemoradiotherapy [35,36,37,38]. Thus, the primary objective of the current study is to analyze the performance of the OSNA assay versus H&E exclusively in RC patients. Our secondary objective is to determine the prognostic relevance of OSNA results in RC patients.

## 2. Materials and Methods

This prospective, observational and multicenter study was conducted according to the STROBE guidelines [39] and adhered to Helsinki Declaration standards. The institutional ethics committee of Hospital Clinic Barcelona approved this study (HCB/2012/7324, on March 2012). All samples were processed pseudo-anonymously.

### 2.1. Eligibility of Patients

The study was performed from June 2010 to March 2024 across fifteen participating tertiary hospitals under the umbrella of a population-based CRC screening program. Inclusion criteria were histologically confirmed RC patients over 18 years-old, who underwent curative intended surgical treatment without neoadjuvant therapy. Preoperative MRI/CT imaging confirmed early-stage RC diagnosis in all cases. All tumors were positive for CK19 immunohistochemistry (IHC). Patients with metastatic disease, synchronous carcinomas, familial adenomatous polyposis syndrome, stent-type intraluminal devices, inflammatory bowel disease-related tumors, or the presence of other malignancies were excluded.

### 2.2. Lymph Node Processing and Examination

LNs were freshly dissected from the mesorectal fat by pathologists or experienced technicians within 45 min following surgery. All freshly procured LNs were analyzed by both H&E and OSNA. LNs were bisected along the long axis. Depending on the LN size, half of the LN or a central 1-millimeter-thick slice were formalin-fixed paraffin-embedded (FFPE) for subsequent H&E analysis and conventional pN staging. The rest of the LN was analyzed with the OSNA assay using the pooling method, as described by Rakislova et al. [40]. Following fresh LNs dissection, the surgical specimen underwent standard pathological processing. In some cases, additional LNs were identified after formalin fixation, usually situated within the fat close to the colorectal wall, which were exclusively analyzed by conventional H&E, and contributed to the final pN stage.

### 2.3. One-Step Nucleic Acid Amplification Assay (OSNA)

The OSNA assay is a standardized, quantitative, rapid, and cost-effective technique for LN molecular analysis, which has been previously described in detail [41]. It uses the reverse transcription loop-mediated isothermal amplification (RT-LAMP) at 65 °C with the RD-100i system (Sysmex, Kobe, Japan) to amplify CK19 mRNA. Unlike RT-PCR, no mRNA extraction or purification is required for the process. LN assessment using the OSNA assay was conducted in accordance with the manufacturer’s manual. The OSNA results were obtained in 20–40 min and expressed as the total tumor load (TTL), defined as the sum of CK19 mRNA copies/µL in all LNs from a surgical specimen [40]. The threshold for OSNA positivity is defined as 250 copies/μL [23]. A TTL of ≥6000 copies/μL has been associated with worse DFS and OS in CRC patients [26], thus being considered clinically relevant in this study.

### 2.4. CK19 Immunohistochemistry

CK19 immunohistochemistry was performed on all primary RC to exclude CK19-negative cases. The standard CK19 IHC protocol was used, as described in our previous study [42]. Positive IHC was defined as the presence of membranous staining, with or without cytoplasm staining, in at least 10% of cancer cells.

### 2.5. Estimation of Sample Size

The sample size was calculated using PASS software (version 2021, NCSS LLC, East Kaysville, UT, USA). The calculation was based on the previously reported performance of OSNA assay with a sensitivity of 90% and specificity 96% [43,44], and a 32.5% prevalence of stage III RC derived from 13,160 stage I-III RC patients in the SEER (Surveillance, Epidemiology, and End Results) registry, assuming a two-sided significance level of 0.05, and a statistical power of 90%. The sample size was estimated at 46 patients, including 31 pN0 cases.

### 2.6. Statistical Analysis

Continuous variables were reported as medians (interquartile ranges, IQR), using Mann–Whitney U tests for two-group comparisons or Kruskal–Wallis H tests for multi-group analyses. The comparisons between categorical variables were conducted using chi-square or Fisher’s exact test. Diagnostic test agreement was quantified using Cohen’s kappa coefficient, while the marginal homogeneity of paired nominal data was analyzed via McNemar’s test. Binary logistic regression analysis was used to investigate risk factors associated with nodal tumor load of TTL ≥ 6000 copies/μL assessed by OSNA. A Cox proportional hazards model was used to estimate hazard ratios (HRs) with 95% confidence intervals (CIs). Given the low event rate in this study, both logistic and Cox regression analyses were conducted using Firth’s penalized likelihood method [45,46]. Kaplan–Meier (KM) curve with log-rank tests were performed to examine differences in CSS (an interval from surgical intervention to cancer-related death) and RFS (an interval from surgical intervention to cancer relapse). Restricted cubic spline regression (RCS) models with three knots were performed to investigate potential nonlinear relationships between log-transformed TTL and HRs for CSS or RFS in RC patients. A *p*-value of <0.05 was regarded as statistically significant. All analyses were performed using the R program (Version 4.2.2).

## 3. Results

### 3.1. Patient Characteristics

This prospective multicenter study included 721 patients with pathologically con-firmed stage I-III CRC (AJCC 8th edition) from fifteen centers. Finally, 671 patients met the inclusion criteria and were included, comprising 97 RC and 574 CC patients, respectively (Table S1). All clinicopathological features were well-balanced between RC and CC patients, except for tumor grade, with a higher prevalence of low-grade tumors among RC patients (79.4% vs. 64.6%, *p* = 0.004). RC patients exhibited a trend of improved 3-year OS/DFS/RFS compared to CC patients, though statistical significances were not reached (Table 1).

A total of 2067 LNs were dissected from 97 RC patients, comprising 1719 freshly dissected LNs and 348 LNs obtained following formalin fixation. In 46 patients, all LNs were assessed by OSNA assay (range 3–62, median 18.0). In the remaining 51 cases, a range of 1–34 post-fixation LNs were identified, with the OSNA assay exclusively restricted to freshly dissected LNs (range 5–39, median 13.0).

### 3.2. Diagnostic Performance of OSNA Versus H&E

**Figure 1:**
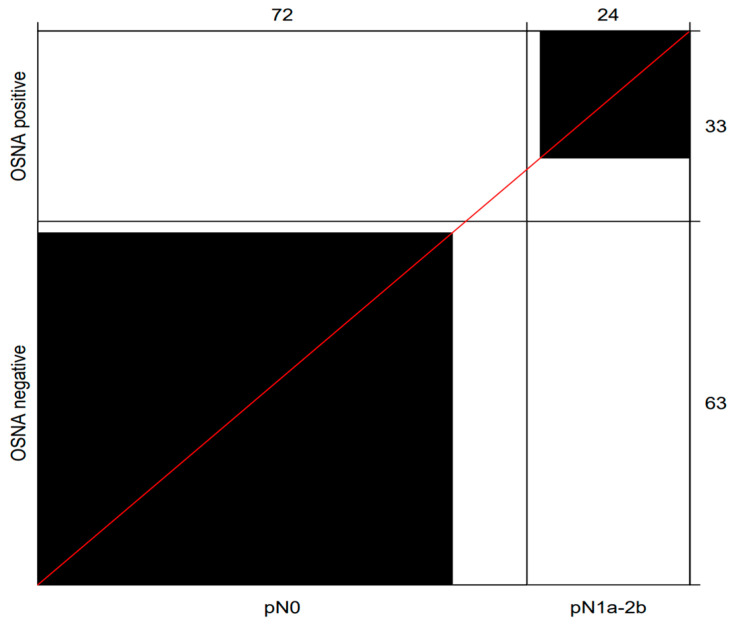
Bangdiwala’s Observer Agreement Chart illustrating the diagnostic performance of OSNA versus H&E in the assessment of nodal metastases. The black boxes indicate the concordant cases. Of the 24 positive cases assessed by H&E, 22 cases were also positive with OSNA. A total of 61 out of 72 cases determined to be negative by H&E were also negative with OSNA. Abbreviation: OSNA, one-step nucleic amplification assay.

The OSNA assay up-staged 15.3% (11/72) of RC patients originally assessed as node-negative on H&E. These up-staged cases exhibited TTL values between 320 and 38,370 copies/μL, with 2 cases exceeding the threshold of 6000 copies/μL associated with worse prognosis. Among the 24 H&E-positive patients, 22 were also positive with OSNA. Overall, the sensitivity, specificity, and negative predictive value of OSNA analysis, in contrast to H&E, were 0.917 (95% CI: 0.730–0.990), 0.847 (95% CI: 0.743–0.921), and 0.968 (95% CI: 0.882–0.985), respectively (Table 2, Figure 1).

### 3.3. Association Between TTL and Clinicopathological Characteristics

**Figure 2:**
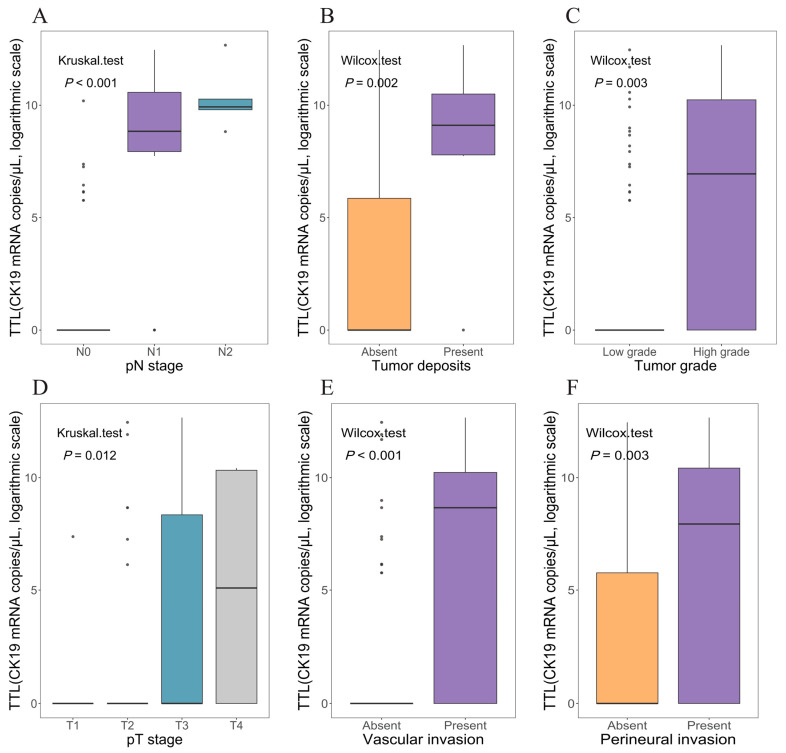
The distribution of TTL values assessed by OSNA across several important pathological factors in rectal cancer patients. Abbreviations: OSNA, one-step nucleic amplification assay; TTL, total tumor load (cytokeratin 19 mRNA copies/μL).

Ninety patients were included in the analysis, with seven excluded for missing values in key variables of interest. Figure 2B–F shows the significant associations of several high-risk features, e.g., the presence of tumor deposits [TDs], perineural invasion [PNI], vascular invasion [VI], G3/4 histology, and pT3/4, with elevated TTL (*p* < 0.05). Notably, TTL significantly increased following pN stages, progressively from pN0 (mean 476.3 copies/μL) to pN1 (mean 53,877.6 copies/μL) and peaking at pN2 (mean 78,420.0 copies/μL) (Figure 2A).

Univariate analysis denoted that the presence of all abovementioned high-risk characteristics was associated with TTL ≥ 250 or ≥6000 copies/μL (*p* < 0.05), as shown in Tables S2 and S3. A Firth-corrected multivariate logistic regression analysis revealed that only high tumor grade (OR = 3.95, 95% CI: 1.01–16.70) and VI+ (OR = 7.65, 95% CI: 1.66–39.05) were independent risk factors for TTL ≥ 6000 copies/μL (Table S3).

### 3.4. Prognostic Relevance of TTL

Seven RC patients (7.2%) were excluded for having follow-up of less than one month. Six extra cases were excluded due to death unrelated to cancer, resulting in 84 patients in the final analysis. The average follow-up was 61.7 months (SD ± 29.0; range 3.8 to 109.5 months). Eight patients (9.5%, 8/84) developed recurrence, with a mean time to recurrence of 13.4 months. The recurrence rates were 3.4% (2/58), 16.7% (2/12), and 28.6% (4/14) for patients with a TTL of <250, 250–6000, and ≥6000 copies/μL, respectively. Patients with recurrence harbored a mean TTL that was 5.7-fold higher (60,187.5 copies/μL) than that of non-recurrent patients (10,474.5 copies/μL). Cancer-specific death occurred in 5 patients (6.0%, 5/84), with a mean time to death of 34.0 months.

**Figure 3:**
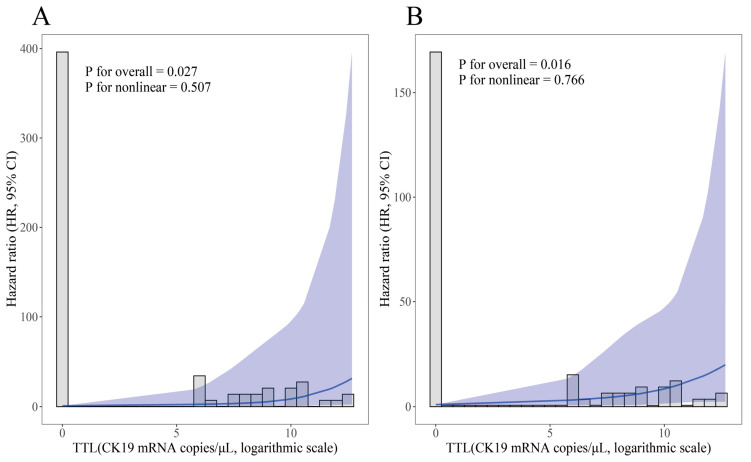
Unadjusted restricted cubic spline regression analyses of relationships between log-transformed TTL value and HRs for cancer-specific survival (**A**) and recurrence-free survival (**B**) in rectal cancer patients. The solid lines represent the estimated hazard ratios, with shaded areas denoting the 95% confidence intervals. Abbreviation: TTL, total tumor load (cytokeratin 19 mRNA copies/μL); HR, hazard ratio.

RCS analyses demonstrated linear dose–response relationships between log-transformed TTL and cancer-specific mortality (*p* for nonlinear = 0.507) and tumor relapse (*p* for nonlinear = 0.766) in RC patients (Figure 3). Then, both TTL ≥250 and ≥6000 copies/μL were examined to ascertain their potential prognostic significance in RC patients. Univariate Cox analysis using Firth’s penalized likelihood method revealed that TTL ≥6000 copies/μL was significantly associated with worse CSS (HR = 6.90, 95% CI: 1.17–40.85) and RFS (HR = 5.34, 95% CI: 1.33–21.39), in contrast to a TTL of <6000 copies/μL. When TTL was trichotomized at 250 and 6000 copies/μL, a TTL of ≥6000 copies/μL conferred a 9.07-fold increased risk of cancer-related death (95% CI: 1.02–80.26) and a 7.90-fold elevated rate of cancer relapse (95% CI: 1.46–42.63), in contrast to a TTL < 250 copies/μL. TTL ≥ 250 versus <250 copies/μL exhibited a significantly association with worse RFS (HR = 5.86, 95% CI: 1.25–27.52); however, when TTL was trichotomized, TTL levels within the range of 250–6000 copies/μL versus <250 copies/μL conferred no worse prognosis to RC patients (Table 3).

**Figure 4:**
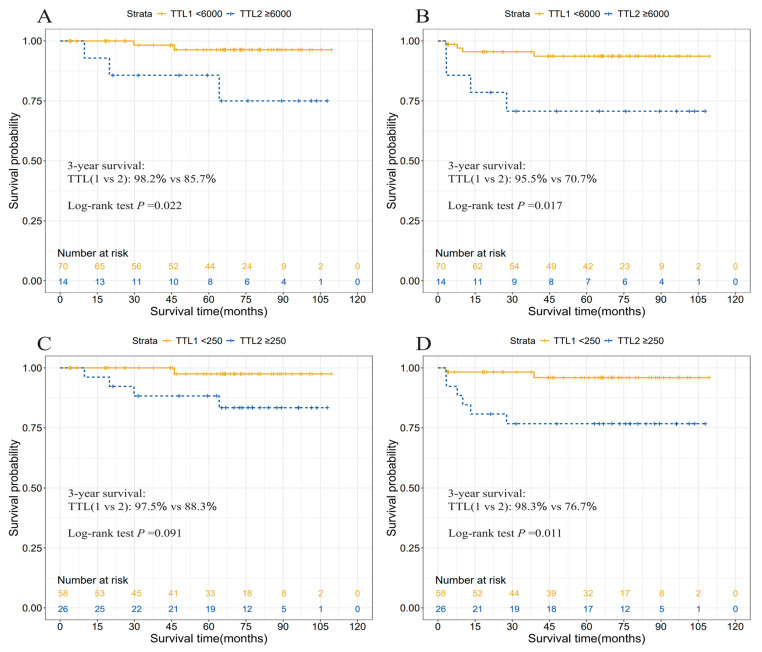
Cancer-specific survival (**A**,**C**) and recurrence-free survival (**B**,**D**) curves according to two TTL subgroups divided by 250 or 6000 copies/μL in rectal cancer patients. Abbreviations: TTL, total tumor load (cytokeratin 19 mRNA copies/μL).

**Figure 5:**
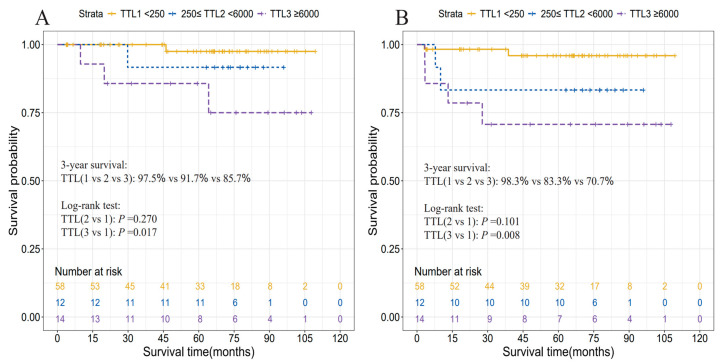
Cancer-specific survival (**A**) and recurrence-free survival (**B**) curves according to three TTL subgroups divided by 250 and 6000 copies/μL in rectal cancer patients. Abbreviation: TTL, total tumor load (cytokeratin 19 mRNA copies/μL).

KM curves illustrated a significantly reduced 3-year CSS and RFS associated with TTL ≥ 6000 copies/μL, in contrast to a TTL of <250 or 6000 copies/μL (Figure 4 and Figure 5).

## 4. Discussion

This multicenter prospective observational study aimed to investigate the performance of the OSNA assay in detecting LNM and its prognostic relevance in RC patients. The results presented here demonstrated that the OSNA assay exhibited higher sensitivity in detecting nodal metastases than H&E, and that a TTL of ≥6000 copies/μL was indicative of worse CSS and RFS in RC patients. Thus, incorporating the OSNA assay into the management of non-metastatic RC may optimize diagnostic accuracy and therapeutic decision-making as it has already been applied in breast cancer in routine clinical practice [47], and its diagnostic accuracy has proved to be as good as conventional histological methods in other solid neoplasms, such as papillary thyroid carcinoma [48] or gastric carcinoma [49].

A burgeoning consensus suggest that the term “CRC” should be abandoned as a single entity in research. Concerning OSNA, studies have been predominantly focused on CRC as a whole [26,27,40,41,50,51] or on CC [52,53]. To the best of our knowledge, our study represents the first to specifically focus on RC patients. Our RC cohort had similar clinicopathological characteristics to CC patients, except for tumor grade. In the past, RC patients have been regarded to have worse prognosis than CC patients. Nevertheless, several advances in treatment have improved the prognosis of RC over the years, including the advent of TME [54], the standard use of neoadjuvant therapy in stages II-III RC [55,56], as well as the popularity of total neoadjuvant treatment [57]. Several studies have revealed that the prognosis of RC is not worse or even slightly better than that of CC, with a 5-year net survival ranging between 61.6 and 70.9% for RC compared to 59.1 and 70.9% for CC [35,36,37,38,58]. Our findings also support this contention. Yet, a recent study using multivariate analysis concluded that CC demonstrated better survival outcomes than RC [59]. The role of postoperative aCT remains controversial in stages II-III RC, especially following preoperative chemoradiotherapy and TME. Some studies refuted the usefulness of aCT [60,61]. Others supported its use in a fraction of RC patients rigorously selected according to a risk-stratified algorithm and informed patient preferences [62,63], whereas a growing body of evidence unequivocally favored the offer of aCT, even for stage II RC [64,65,66,67].

As stated above, the inconsistent results in trials render aCT one of the most controversial issues in RC. Notably, the gains of aCT are not so pronounced in RC versus CC, likely due to a delay from diagnosis to aCT initiation caused by preoperative treatment [62]. In non-pretreated RC patients, aCT should be administered just like in CC patients [62]. In these patients with stages I-III RC, a significantly reduced risk of death or relapse was observed following aCT [64]. Moreover, the effect of aCT on both death and recurrence in RC was greater, albeit not significantly, than that in CC, with a similar pattern observed when comparing stage II diseases [64]. LN status determines the use of aCT following surgery in CRC patients [8]. As a simple, fast, and automated technique with a performance comparable to the histopathological examination at 2-millimeter intervals [23], OSNA could be helpful to identify clinically relevant LN involvements in a subset of pN0 patients who might benefit from aCT or intensive surveillance. Moreover, as it has been established that high-volume hospitals generally guarantee a better quality in the application of certain techniques, one of the main advantages of the OSNA assay is that it allows an automated and standardized RT-PCR in a short time without the need for advanced technical expertise, thus enabling its use in small centers [68].

The previously reported upstaging rate of the OSNA assay in CRC patients ranged from 11.3% to 51.0% [24,40,42,69]. In the present study, the OSNA assay up-staged 15.3% of RC patients with negative H&E results, which underscored the potential of the OSNA assay in identifying clinically relevant LN involvement in pN0 RC patients. The OSNA assay identified 22 of 24 H&E-positive patients, resulting in a sensitivity of 91.7%. The false-negative OSNA results in two patients, each with one H&E-positive LN, were likely attributed to tumor allocation bias (TAB). Overall, our findings indicated that the diagnostic ability of OSNA in RC was in line with prior results observed in CRC [43,44].

As mentioned above, nodal occult metastases or MMs, detected by IHC or PCR or RT-PCR based techniques, have been linked to adverse prognosis in pN0 CRC patients assessed by H&E [10,11,70]. With improved LN tissue sampling, the OSNA assay targets CK19 mRNA, continuously quantifying nodal tumor load, and bridges true stage I and III tumors by identifying occult nodal metastases in stage II cancers. This was evidenced by a significant increase in TTL with the incremental pN stages, by a large overlap of TTL values between up-staged “stage II” CRC patients and those with stage III disease [24], as well as by a linear dose–response relationship between log-transformed TTL and cancer-specific mortality/tumor relapses in the present study. However, a widely accepted TTL threshold with prognostic relevance has not been established up to now. A prior study revealed that a TTL of ≥250 copies/μL was associated with significantly lower DFS in stage II CRC patients [27]. Conversely, in another study, the authors highlighted that OSNA positivity did not add any prognostic value to conventional histologic survey in patients with stages I-III CC [52]. Moreover, in a prior study, we observed that a relatively low tumor volume between 400 and 4270 copies/μL failed to confer adverse prognosis to patients with in situ CRC within a median follow-up of 49.6 months [51]. In this setting, Archilla et al. demonstrated that a TTL of ≥6000 copies/μL was associated with worse OS and DFS [26]. In their study, the establishment of this threshold was based on a larger sample size of 342 stage I-III CRC patients. Herein, we applied a Cox proportional hazard regression with Firth’s penalized likelihood method to account for the low incidence of cancer death and relapses [46]. The multivariate Cox analysis was not performed due to inadequate events per variable [46]. We concluded that a TTL of ≥6000 copies/μL was related to worse CSS and RFS, which may help select a subset of resectable RC patients with increased risk of cancer recurrence and worse prognosis. However, the benefit of aCT or intensive surveillance for these patients remains to be examined. In contrast, the result of significantly reduced RFS in patients with TTL ≥250 versus <250 copies/μL was likely a false positive, as the limited sample size may have resulted in a minimal variation in patient allocations.

Additionally, VI+, PNI+, TDs+, high tumor grade, and T-stage, were significantly associated with LNM assessed by OSNA, which were in line with prior studies [24,26,41,42]. Whereas only VI+ and high tumor grade were identified as independent predictors for TTL ≥ 6000 copies/μL using multivariate analysis. These clinicopathological factors may help identify high-risk stage II RC patients, although only modest survival benefit from aCT could be conferred to these patients according to a prior study [71].

There are several limitations in the present study. Firstly, LNs were partially assessed by OSNA, and partially by H&E, which may have resulted in an intrinsic TAB. Secondly, the OSNA assay is currently restricted to freshly procured LNs. Hence, a fraction of metastatic LNs may not be freshly obtained and assessed by OSNA, undermining an accurate evaluation of its role in clinical practice. Thirdly, herein, a TTL of ≥6000 copies/μL was assumed to hold prognostic relevance according to a prior study [26], which may be suboptimal. The establishment of such a threshold should be upheld by the whole LN analysis using OSNA, multicenter prospective large-scale studies with extended patient follow-up, as well as the adoption of improved node retrieval techniques. Lastly, a small sample size may contribute to reproducibility issues, thereby introducing uncertainty and compromising the interpretation of results.

## 5. Conclusions

The OSNA assay is highly sensitive for detecting nodal metastases in RC patients compared to H&E analysis. The OSNA results have prognostic implications in RC patients, with a TTL of ≥6000 copies/μL related to worse CSS and RFS. This threshold could be helpful in decision-making, especially in the selection of candidates for aCT or intensive surveillance. Future directions should include large-scale studies to validate our findings. Furthermore, the entire LNs should be analyzed with OSNA, rendering a more accurate evaluation of its role in clinical practice. The latter would allow setting a definite TTL cutoff with a prognostic and predictive value, which would enable better patient management.

## Figures and Tables

**Table 1:** Demographic and clinicopathological characteristics of RC vs. CC patients.

Characteristics	RC Patients (*n* = 97)	CC Patients (*n* = 574)	*p*-Value
Sex (male), *n* (%)	58 (59.8)	344 (59.9)	0.980
Age (years), median (IQR)	67.0 (59.0, 78.0)	71.0 (62.0, 80.0)	0.083
Range (years)	41–90	24–94	
Tumor size (cm), median (IQR)	3.2 (2.1, 4.0)	3.1 (2.0, 4.5)	0.779
Range (cm)	0.3–7.1	0.2–13	
Tumor grade, *n* (%)			0.004
Low (G1–G2)	77 (79.4)	371 (64.6)	
High (G3–G4)	20 (20.6)	203 (35.4)	
Tumor budding, *n* (%) ^†^			0.475
Bd1	68 (73.9)	372 (69.5)	
Bd2	14 (15.2)	108 (20.2)	
Bd3	10 (10.9)	55 (10.3)	
Perineural invasion, *n* (%) ^†^			0.326
Absent	82 (84.5)	503 (88.1)	
Present	15 (15.5)	68 (11.9)	
Vascular invasion, *n* (%)			0.855
Absent	71 (73.2)	415 (72.3)	
Present	26 (26.8)	159 (27.7)	
Tumor deposits, *n* (%) ^†^			0.738
Absent	90 (92.8)	534 (93.7)	
Present	7 (7.2)	36 (6.3)	
Total LNs, median (IQR)	19.0 (15.0, 24.0)	19.0 (15.0, 25.0)	0.719
Fresh LNs, median (IQR)	16.0 (12.0, 21.0)	16.0 (11.0, 20.8)	0.607
Post-fixation LNs, median (IQR)	1.0 (0, 6.0)	2.0 (0, 5.0)	0.292
pT stage, *n* (%)			0.084
T1	18 (18.6)	103 (17.9)	
T2	27 (27.8)	117 (20.4)	
T3	46 (47.4)	277 (48.3)	
T4	6 (6.2)	77 (13.4)	
pN stage, *n* (%)			0.673
N0	72 (74.2)	430 (74.9)	
N1	19 (19.6)	102 (17.8)	
N2	6 (6.2)	42 (7.3)	
pStage, *n* (%)			0.452
I	40 (41.2)	199 (34.7)	
II	32 (33.0)	231 (40.2)	
III	25 (25.8)	144 (25.1)	
TTL, median (IQR)	0 (0, 2300)	0 (0, 1350)	0.993
Range	0–318,000	0–1,724,500	
Mean	15,873.40	20,608.80	
OSNA positive ^a^, *n* (%)	33 (34.0)	205 (35.7)	0.747
OSNA clinically relevant ^b^, *n* (%)	19 (19.6)	87 (15.2)	0.268
Adjuvant chemotherapy, *n* (%) ^†^			0.561
No	75 (77.3)	416 (74.6)	
Yes	22 (22.7)	142 (25.4)	
3-year OS (IQR, %) ^†^	92.3 (86.5, 98.4)	89.9 (87.0, 92.9)	0.364
3-year DFS (IQR, %) ^†^	89.0 (82.4, 96.1)	82.6 (79.0, 86.2)	0.184
3-year RFS (IQR, %) ^†^	93.0 (87.7, 98.6)	86.9 (83.8, 90.2)	0.197

RC, rectal cancer; CC, colon cancer; IQR, interquartile range; TTL, total tumor load (cytokeratin 19 mRNA copies/μL); OSNA, one-step nucleic acid amplification assay; OS, overall survival; DFS, disease-free survival; RFS, recurrence-free survival; LNs, lymph nodes. ^†^, analysis based on a subset of patients due to missing data; ^a^, a TTL value of not less than 250 copies/μL was regarded as positive for nodal metastases; ^b^, a TTL value of not less than 6000 copies/μL was regarded as clinically relevant according to a prior study [26].

**Table 2:** Diagnostic performance of OSNA versus H&E in detecting nodal metastases in rectal cancer patients.

		H&E, pN Stage		*n*	Concordance (95% CI)	Sensitivity (95% CI)	Specificity (95% CI)	PPV (95% CI)	NPV (95% CI)	Kappa Index	McNemar’s *p*-Value
		N0	N1a~2b								
OSNA	−	61	2 ^†^	96	0.865 (0.780, 0.926)	0.917 (0.730, 0.990)	0.847 (0.743, 0.921)	0.667 (0.511, 0.946)	0.968 (0.882, 0.985)	0.679 (0.517, 0.841)	0.013
	+	11	22								

OSNA, one-step nucleic acid amplification; PPV, positive predictive value; NPV, negative predictive value; CI, confidence interval; ^†^, One OSNA-negative patient with 1 positive lymph node, assessed by post-fixation H&E, was excluded.

**Table 3:** Univariate Cox regression analyses according to nodal status assessed by OSNA/H&E in rectal cancer patients.

Nodal Status Assessed by OSNA/H&E	No. of Patients (%)	CSS		RFS	
		HR (95% CI)	*p*-Value	HR (95% CI)	*p*-Value
TTL (logarithmic scale), continuous	84 (100)	1.27 (1.02, 1.56)	0.010	1.24 (1.06, 1.45)	0.003
OSNA positive ^a^					
No	58 (69.0)	ref		ref	
Yes	26 (31.0)	5.72 (0.76, 43.35)	0.091	5.86 (1.25, 27.52)	0.011
OSNA clinically relevant ^b^					
No	70 (83.3)	ref		ref	
Yes	14 (16.7)	6.90 (1.17, 40.85)	0.022	5.34 (1.33, 21.39)	0.017
TTL subgroups, copies/μL					
[0, 250)	58 (69.0)	ref		ref	
[250, 6000)	12 (14.3)	3.73 (0.28, 49.42)	0.270	4.63 (0.68, 31.28)	0.101
[6000, ~)	14 (16.7)	9.07 (1.02, 80.26)	0.017	7.90 (1.46, 42.63)	0.008
pN stage					
N0	63 (75.0)	ref		ref	
N1/2	21 (25.0)	8.66 (1.14, 65.60)	0.012	8.48 (1.80, 39.89)	0.002
pN stage					
N0	63 (75.0)	ref		ref	
N1	16 (19.0)	5.97 (0.59, 60.08)	0.080	5.56 (0.95, 32.59)	0.051
N2	5 (6.0)	24.72 (2.45, 249.61)	0.003	26.58 (4.44, 158.99)	<0.001

CSS, cancer specific survival; RFS, recurrence-free survival; HR, hazards ratio; CI, confidence interval; OSNA, one-step nucleic acid amplification; TTL, total tumor load (cytokeratin 19 mRNA copies/μL); No., number; ref, reference; ^a^, a TTL value of not less than 250 copies/μL was deemed as positive for nodal metastases; ^b^, a TTL value of not less than 6,000 copies/μL was deemed as clinically relevant according to a prior study [26].

## Data Availability

The original contributions presented in this study are included in the article/Supplementary Materials. Further inquiries can be directed to the corresponding authors.

## Supplementary Materials

The following supporting information can be downloaded at: https://www.mdpi.com/article/10.3390/cancers17132141/s1, Table S1: Number of patients provided by each participant center; Table S2: Clinicopathological risk factors associated with OSNA positivity in rectal cancer patients; Table S3: Clinicopathological risk factors associated with TTL > 6000 copies/μL assessed by OSNA in rectal cancer patients.
