# Supplementary material for: Performance and Prognostic Relevance of Lymph Node Assessment by One-Step Nucleic Acid Amplification Assay in Rectal Cancer: A Multicenter Study
Source: Cancers (Basel). 2025 Jun 25;17(13):2141. doi: 10.3390/cancers17132141 (PMC12249117; doi:10.3390/cancers17132141)
Supplement: Supplementary file 1 [file cancers-17-02141-s001.zip › cancers-3707756-supplementary.pdf]

Supplementary Materials

Performance and Prognostic Relevance of Lymph Node Assessment by One-Step Nucleic Acid Amplification Assay in Rectal Cancer: A Multicenter Study

Qing Liu, Sandra Lopez-Prades, Karmele Saez de Gordoa, Maite Rodrigo-Calvo, Mireia Garcia, Juan Ruiz Martin, Angel Romo, Ignacio Pinilla, Jordi Tarragona, Begoña Otero Alen, Jordi Camps, Ivan Archilla and Miriam Cuatrecasas

Table S1. Number of patients provided by each participant center.

Participating Centers	No. of CC patients	No. of RC patients	Period of recruitment
Hospital 1	376	84	2012-2024
Hospital 2	75	2	2010-2023
Hospital 3	21	5	2016-2021
Hospital 4	11	1	2017-2018
Hospital 5	3	4	2012-2013
Hospital 6	15	1	2012-2013
Hospital 7	24	0	2017-2024
Hospital 8	14	0	2012-2013
Hospital 9	7	0	2012-2013
Hospital 10	3	0	2012-2013
Hospital 11	5	0	2022-2023
Hospital 12	6	0	2012-2013
Hospital 13	8	0	2018
Hospital 14	3	0	2012
Hospital 15	3	0	2012

Table S2. Clinicopathological risk factors associated with OSNA positivity in rectal cancer patients.

Characteristics	OSNA negative	OSNA positive[a]	P value
Sex, n (%)			0.813
Male	36 (58.1)	17 (60.7)	
Female	26 (41.9)	11 (39.3)	
Age (years), mean (SD)	68.4 (12.0)	66.8 (13.1)	0.562
Age (years), n (%)			0.951
< 70	35 (56.5)	16 (57.1)	
≥ 70	27 (43.5)	12 (42.9)	
Tumor size (cm), median (IQR)	3.3 (2.0, 4.1)	3.5 (2.5, 4.0)	0.499
Tumor size (cm), n (%)			0.777
< 3.5	33 (53.2)	14 (50.0)	
≥ 3.5	29 (46.8)	14 (50.0)	
Grade, n (%)			**0.009**
Low (G1, G2)	53 (85.5)	17 (60.7)	
High (G3, G4)	9 (14.5)	11 (39.3)	
Tumor budding, n (%)			0.159
Bd1	49 (79.0)	18 (64.3)	
Bd2	7 (11.3)	6 (21.4)	
Bd3	6 (9.7)	4 (14.3)	
Perineural invasion, n (%)			**0.025**
Absent	57 (91.9)	20 (71.4)	
Present	5 (8.1)	8 (28.6)	
Vascular invasion, n (%)			**< 0.001**
Absent	55 (88.7)	12 (42.9)	

Present	7 (11.3)	16 (57.1)	
Tumor deposits, n (%)			**0.016**
Absent	61 (98.4)	23 (82.1)	
Present	1 (1.6)	5 (17.9)	
pT stage, n (%)			**0.014**
T1	16 (25.8)	1 (3.6)	
T2	21 (33.9)	6 (21.4)	
T3	22 (35.5)	18 (64.3)	
T4	3 (4.8)	3 (10.7)	

OSNA, one-step nucleic acid amplification; TTL, total tumor load (cytokeratin 19 mRNA copies/μL); IQR, interquartile range; SD, standard deviation. a, a TTL value of not less than 250 copies/μL was regarded as positive for nodal metastases.

Table S3. Clinicopathological risk factors associated with TTL >6000 copies/μL assessed by OSNA in rectal cancer patients.

Characteristics	No. of patients (%)	Univariate analysis		Multivariate analysis	
		OR (95% CI)	P value	OR (95% CI)	P value
Sex					
Male	53 (58.9)	ref			
Female	37 (41.1)	1.78 (0.60, 5.42)	0.294		
Age, continuous	90 (100)	1.00 (0.95, 1.04)	0.904		
Age (years), categorical					
< 70	51 (56.7)	ref			
≥70	39 (43.3)	1.18 (0.39, 3.51)	0.763		
Tumor size, continuous	90 (100)	1.28 (0.91, 1.82)	0.154		
Tumor size (cm), categorical					
< 3.5	47 (52.2)	ref			
≥3.5	43 (47.8)	1.29 (0.44, 3.90)	0.641		
Grade					
Low (G1, G2)	70 (77.8)	ref		ref	
High (G3, G4)	20 (22.2)	5.76 (1.82, 18.85)	**0.003**	3.95 (1.01, 16.70)	**0.049**
Tumor budding					
Bd1	67 (74.4)	ref			
Bd2	13 (14.4)	1.83 (0.41, 6.87)	0.406		
Bd3	10 (11.1)	1.61 (0.28, 6.97)	0.560		
Perineural invasion					
Absent	77 (85.6)	ref		ref	
Present	13 (14.4)	6.25 (1.77, 22.35)	**0.005**	0.91 (0.15, 4.56)	0.914
Vascular invasion					
Absent	67 (74.4)	ref		ref	
Present	23 (25.6)	12.98 (3.95, 49.89)	**<0.001**	7.65 (1.66, 39.05)	**0.009**
Tumor deposits					
Absent	84 (93.3)	ref		ref	
Present	6 (6.7)	5.80 (1.11, 30.50)	**0.038**	0.94 (0.12, 7.35)	0.949
pT stage					
T1	17 (18.9)	ref		ref	
T2	27 (30.0)	3.43 (0.26, 484.67)	0.383	5.85 (0.34, 967.52)	0.247
T3	40 (44.4)	12.05 (1.39, 1584.40)	**0.019**	10.56 (0.84, 1628.10)	0.072
T4	6 (6.7)	35.00 (2.59, 5140.90)	**0.005**	11.36 (0.41, 2093.07)	0.157

OSNA, one-step nucleic acid amplification; TTL, total tumor load (cytokeratin 19 mRNA copies/μL); OR, odds ratio; CI, confidence interval; No., number; ref, reference.
